# Exercise training outcomes in patients with chronic heart failure with reduced ejection fraction depend on patient background

**DOI:** 10.3389/fcvm.2024.1330235

**Published:** 2024-01-31

**Authors:** Yukako Soejima, Hideki Yoshioka, Sayuri Guro, Hiromi Sato, Hiroto Hatakeyama, Yasunori Sato, Yoshihide Fujimoto, Naohiko Anzai, Akihiro Hisaka

**Affiliations:** ^1^ Clinical Pharmacology and Pharmacometrics, Graduate School of Pharmaceutical Sciences, Chiba University, Chiba, Japan; ^2^ Early Development, Astellas Pharma Inc., Tokyo, Japan; ^3^ Department of Preventive Medicine and Public Health, Keio University School of Medicine, Tokyo, Japan; ^4^ Department of Cardiology, International University of Health and Welfare Narita Hospital, Chiba, Japan; ^5^ Department of Pharmacology, Graduate School of Medicine, Chiba University, Chiba, Japan

**Keywords:** exercise training, chronic heart failure, HF-ACTION, risk factors, Cox regression analysis, Boruta method, predictive scores

## Abstract

**Background:**

The aim of this study was to identify significant factors affecting the effectiveness of exercise training using information of the HF-ACTION (Heart Failure: A Controlled Trial Investigating Outcomes of Exercise Training) study.

**Methods:**

Background factors influencing the effect of exercise training were comprehensively surveyed for 2,130 patients by multivariable Cox regression analysis with the stepwise variable selection, and only significant factors were selected that were statistically distinguished from dummy noise factors using the Boruta method.

**Results:**

The analysis suggested that the use of beta-blockers, pulse pressure, hemoglobin level, electrocardiography findings, body mass index, and history of stroke at baseline potentially influenced the exercise effect on all-cause death (AD). Therefore, a hypothetical score to estimate the effect of exercise training was constructed based on the analysis. The analysis suggested that the score is useful in identifying patients for whom exercise training may be significantly effective in reducing all-caused death and hospitalization (ADH) as well as AD. Such a subpopulation accounted for approximately 40% of the overall study population. On the other hand, in approximately 45% of patients, the effect of exercise was unclear on either AD or ADH. In the remaining 15% of patients, it was estimated that the effect of exercise might be unclear for ADH and potentially rather increase AD.

**Conclusions:**

This study is the first analysis to comprehensively evaluate the effects of various factors on the outcome of exercise training in chronic heart failure, underscoring the need to carefully consider the patient's background before recommending exercise training. However, it should be noted that exercise training can improve many outcomes in a wide variety of diseases. Therefore, given the limitations involved in *post-hoc* analyses of a single clinical trial, the characteristics of patients to whom the results of this analysis can be applied need attention, and also further research is necessary on the relationship between the degree of exercise and the outcomes. A new clinical trial would be needed to confirm the factors detected and the appropriateness of the score.

## 1 Introduction

Exercise training improves mortality, health-related quality of life, and exercise capacity in patients with chronic heart failure (1–6) and is widely recommended as an effective treatment that can be combined with pharmacotherapy such as renin-angiotensin system inhibitors (RASIs, i.e., angiotensin-converting enzyme inhibitor and/or angiotensin II receptor blocker) and beta-blockers (BBs) (7, 8). The Heart Failure: A Controlled Trial Investigating Outcomes of Exercise Training (HF-ACTION) study conducted in 2003–2008 is the only large-scale randomized controlled trial that examined the efficacy of exercise training in medically stable patients with chronic heart failure (9). The trial demonstrated that a prescribed exercise training program was associated with a reduction in all-cause death and hospitalization (ADH) rate. This improvement was statistically significant after the adjustment for prognostic factors, and a non-significant reduction in all-cause death (AD), one of the secondary endpoints, was noted regardless of factor adjustment.

In chronic heart failure, reducing the cardiac load is essential for successful treatment (10–12). We previously performed a model-based meta-analysis of 61 studies in patients with chronic heart failure and reported that estimated myocardial oxygen consumption, a cardiac load index, correlated excellently with the reduction in mortality after various pharmacotherapies (13). In particular, RASIs and BBs reduced the estimated myocardial oxygen consumption more efficiently than other drug classes, including calcium channel blockers and direct renin inhibitors, which supported the superior prognostic improvement ability of these drugs. In contrast, exercise activates the sympathetic nervous system (14) and increases cardiac load, which contradicts the expected effect of drug therapy (15–17). Therefore, the use of RASIs and BBs may affect exercise training efficacy. The subgroup analysis of the HF-ACTION trial showed that various factors including baseline RASI and BB use did not affect exercise effects. However, assuming that a variety of factors including cardiopulmonary capacity, medical condition, and medical history (18–20) potentially affect exercise effects and that these factors are correlated with each other, there would be limitations in revealing the heterogeneity of exercise effects by simple subgroup analyses.

This study, as a *post hoc* analysis of individual patient data from the HF-ACTION trial, aimed to hypothetically elucidate the association between various patient characteristics at baseline and the exercise training effects and provide useful information for individualization of exercise training to optimize benefit of patients.

## 2 Methods

### 2.1 Study population

The HF-ACTION study was a randomized multicenter trial that evaluated the effectiveness of exercise training vs. usual care in patients with chronic heart failure. The study included patients with stable heart failure with a left ventricular ejection fraction (LVEF) of less than 35% and a New York Heart Association (NYHA) class of II–IV despite optimal heart failure treatment for at least 6 weeks. See the original report for HF-ACTION study for details including exercise training procedures (9).

### 2.2 Outcomes

The primary endpoints in this analysis were (1) AD; and (2) ADH during the entire follow-up period (up to 4 years; median, 30 months). We adopted an intention-to-treat population analysis.

### 2.3 Variables

Variables missing in more than 30% of the samples were excluded beforehand and remaining missing data were complemented by a multiple imputation method (21). To avoid obvious multicollinearity, Spearman's rank correlation coefficient of greater than 0.8 for continuous variables and the chi-square value of greater than 300 for categorical variables were excluded. The resulting 73 variables (Supplementary Table S1) were used to in the subsequent Cox analysis to comprehensively explore the interaction effects with exercise training. All continuous variables were transformed into binary variables nearly at the 25th, 50th, and 75th percentiles.

### 2.4 Cox proportional hazards models

Main effects of each variable and their interaction with exercise training was tested with a univariate Cox analysis, and those with a *P*-value of less than 0.3 were selected. The stepwise method with a significance level of *P* = 0.1 for inclusion and of *P* = 0.05 for exclusion was then applied to the selected variables to develop a full model.

To exclude effects with a potentially high false positive rate (FDR) and obtain a robust final model, we applied the Boruta method to the full model (22). Boruta is a variable selection approach commonly used in the area of machine learning/artificial intelligence, which attempts to select meaningful variables by intentionally adding meaningless variables to the model. Briefly, Boruta first prepares an equal number of dummy variables as candidate real variables. These dummy variables are copies of the real variables, but shuffled in the sample direction and should therefore have no importance in the predictive model. A model is then developed using data containing both real and dummy variables, and the real variables that are more important than the dummy variables are selected. This is repeated N times with a certain degree of randomness. If the importance of a real variable is the same as the corresponding dummy variable (i.e., it is meaningless), the probability that it is selected should be 50%, so the number of times a real variable is selected follows a binomial distribution with *p* = 0.5 and *n* = N. Finally, whether the number of times the real variable is selected is sufficiently large is tested based on this binomial distribution at any significance level α. In this analysis, we applied Boruta to each of the main effects and interaction effects in the full model using 1,000 datasets generated by the bootstrap method and excluded effects with FDR greater than 0.05 (i.e., *N* = 1,000, *α* = 0.05). The -log(p) value was used as the importance measure of the variables, and the 95th percentile value of the importance of the dummy variables was used as the threshold for the selection of important real variables. To avoid population imbalance, the bootstrapping was performed by stratifying data by the treatment group, medication group, and death or hospitalization at 4 years.

In the series of variable selection processes, the interaction between the choice of medication at baseline and exercise training was always accounted for by modeling the exercise effect separately for each medication group. The medication was categorized into three groups according to the use of BBs and RASIs at baseline: both BB and RASI, RASI only, and BB only. The exercise effect in the subgroup of patients who were not treated with neither RASI nor BB at baseline was neglected because the sample size was too small (*n* = 10; 0.5%). Angiotensin-converting enzyme inhibitors (ACEIs) and angiotensin II receptor blockers (ARBs) were combined as RASIs. The interaction *P*-value for the medication group and exercise training was assessed posteriori by a likelihood ratio test comparing models with and without splitting the exercise effect according to medication.

To ensure the interpretability of the effects of the exercise training in the final model, all the variables other than the exercise effect were zero-centered before the Cox regression analysis (i.e., subtracting the mean of each variable). This process sets the baseline hazard to the overall mean of the usual care subjects and avoids the main effect of exercise training being estimated as a value in an extremely biased small population without changing the statistical significance of the analysis.

### 2.5 Scores predicting benefit or harm from exercise training

Based on the results of the Cox proportional hazards model, the predictive score for exercise eligibility was developed incorporating significant interaction terms identified in the final model. For ease of use, all predictors comprising the score were assigned integer points by scaling of the β-coefficients of the final Cox model. The score was designed such that the higher the total score, the more beneficial exercise training would be.

### 2.6 Software

The PHREG procedure implemented in SAS® 9.4 was used to conduct the Cox proportional hazards model analysis. In addition to SAS, Python 3.8 (Python Software Foundation) was used for the data processing and illustrations. IterativeImputer, implemented in scikit-learn 0.24.2, was used for multiple imputations of missing data.

## 3 Results

### 3.1 Model development

All records of eligible patients from the HF-ACTION trial (2,130 of 2,331 patients consented to share for non-commercial uses and allowed for any research purpose) were included in the analyses.

**Figure 1:**
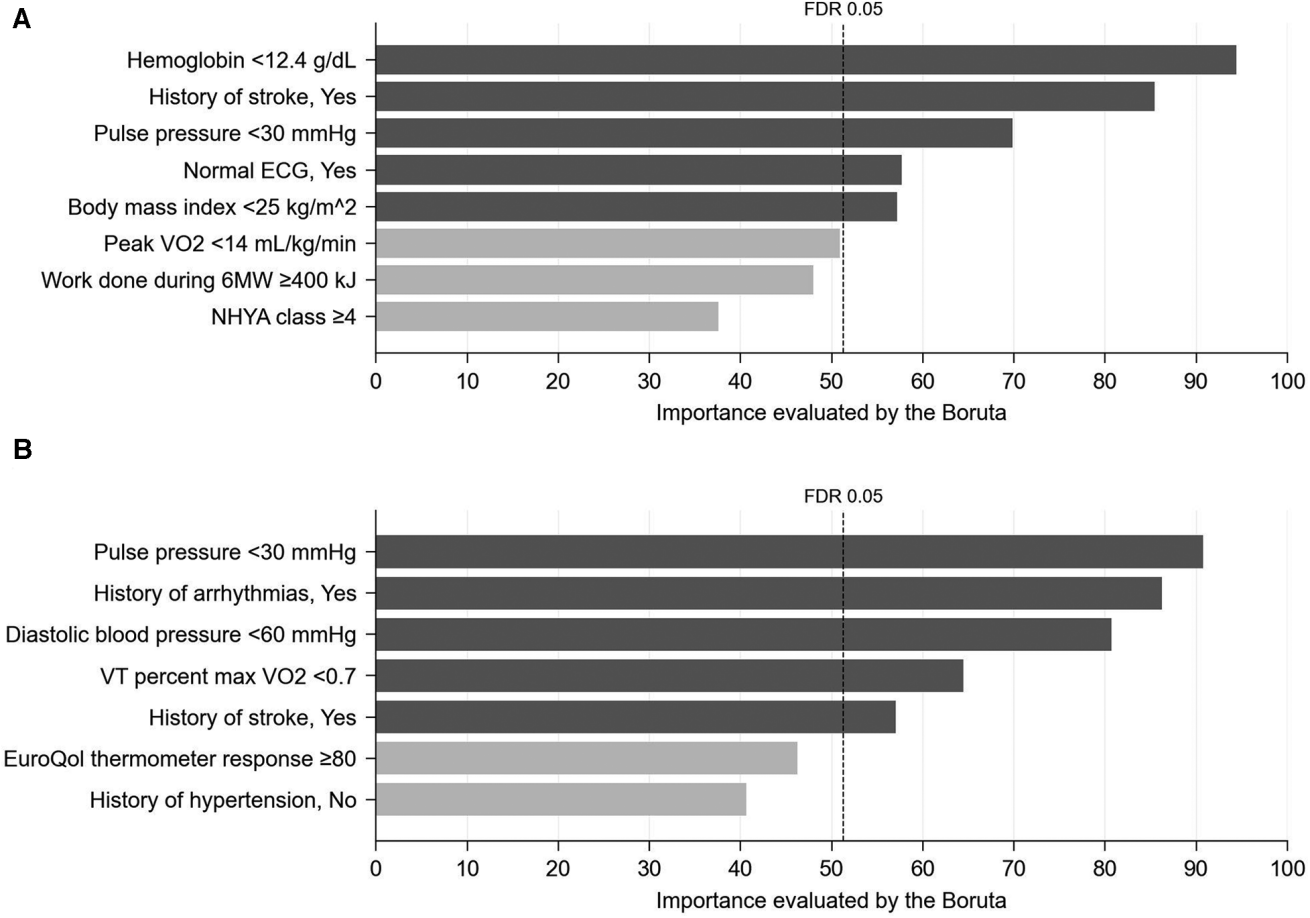
Importance (probability of selection, %) of interaction effects with exercise training assessed using 1,000 bootstrap datasets by the Boruta method (see methods for details). (**A**) AD, (**B**) ADH. FDR, false discovery rate.

As a result of tentative variable selection using the stepwise method, 32 variables as main effects and 8 variables as interactions with exercise training included in the full model for AD, and 39 variables as main effects and 7 variables as interactions with exercise training were included in the full model for ADH. Of these, 6 main effects and 5 interaction effects in the AD model and 17 main effects and 5 interaction effects in the ADH model were considered significant because the FDR calculated by Boruta was smaller than 0.05 (Figure 1 and Supplementary Figure S1; see also Methods for details).

### 3.2 Model interpretation

The overall hazard ratios (HRs) of the exercise effect calculated from the final Cox models were 0.92 (95% CI, 0.81–1.04) for AD and 0.88 (95% CI, 0.82–0.94) for ADH, which was consistent with the original analysis of the HF-ACTION trial reporting that exercise training significantly reduced the risk of ADH with adjustment but not that of AD (9).

**Figure 2:**
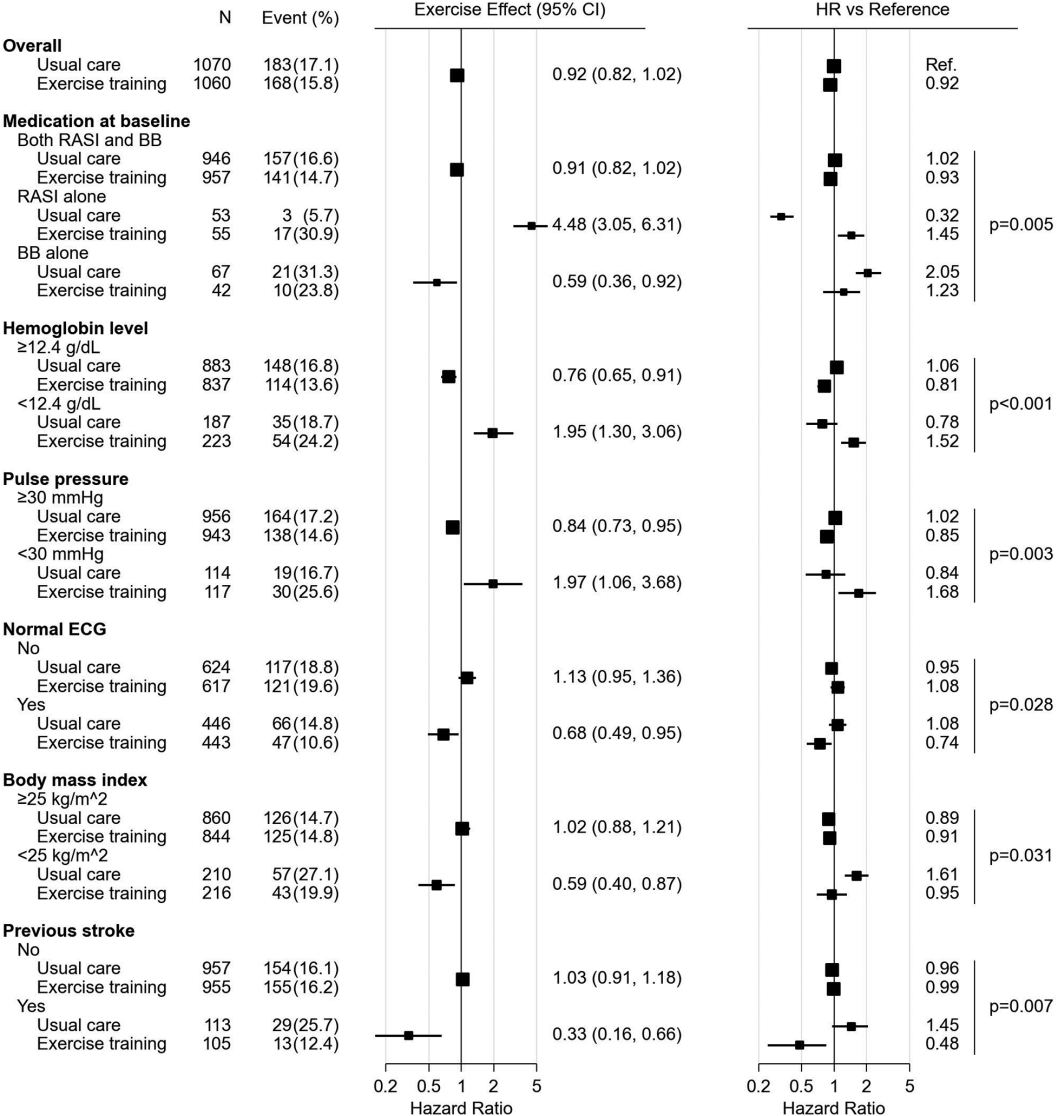
The forest plot represents influence of medication and other characteristics on exercise effect for all-cause death. All the HRs, effects and their CIs were calculated from the analysis of 1,000 bootstrap datasets based on the multivariate Cox proportional hazards model analysis. The hazard ratios of subgroups are presented versus the overall mean of the usual care subjects. The marker size is calculated to be proportional to the square root of the number of subjects. BB, beta-blocker; CI, confidence interval; ECG, electrocardiogram; HR, hazard ratio; RASI, renin-angiotensin system inhibitor.

**Figure 3:**
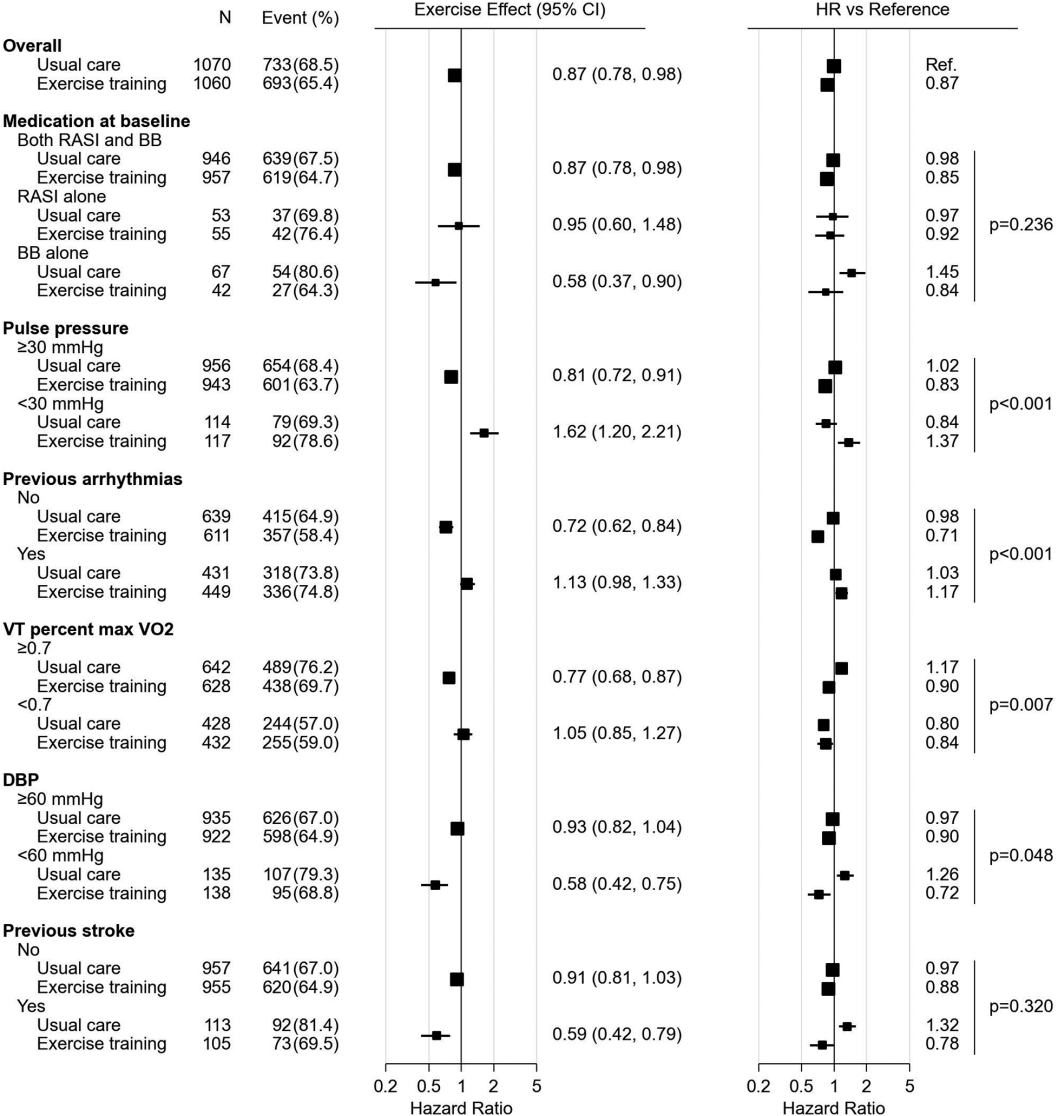
The forest plot represents influence of medication and other characteristics on exercise effect for all-cause death or hospitalization. All the HRs, effects and their CIs were calculated from the analysis of 1,000 bootstrap datasets based on the multivariate Cox proportional hazards model analysis. The figure was prepared in the same format as Figure 1. BB, beta-blocker; CI, confidence interval; ECG, electrocardiogram; HR, hazard ratio; RASI, renin-angiotensin system inhibitor; VO_2_, oxygen uptake.

Forest plots, Figures 2, 3, summarize the significant influence of various patient characteristics at baseline on the effectiveness of exercise training identified by the multivariate Cox proportional hazards model for AD and ADH, respectively. The effects of exercise on AD differed significantly depending on medication at baseline (interaction *P* = 0.005, Figure 2). A tendency toward beneficial exercise effects was estimated in the subgroups taking BBs at baseline, i.e., in patients taking BBs alone (HR, 0.59; 95% CI, 0.36–0.92) and in patients taking both RASIs and BBs (HR, 0.91; 95% CI, 0.82–1.02), whereas exercise training was potentially associated with an increased risk of AD in patients not treated with BBs at baseline, i.e., taking RASIs alone (HR, 4.48; 95% CI, 3.05–6.31). For ADH, on the other hand, the interaction between exercise training and medication at baseline was not statistically significant (interaction *P* = 0.236, Figure 3). For both endpoints, five factors other than medication were identified as significantly interacting with exercise training. Interactions of pulse pressure of <30 mmHg and history of stroke with exercise training were identified in both AD and ADH, with the former associated with adverse exercise effects and, conversely, the latter with beneficial exercise effects. Other factors associated with increased exercise effectiveness included a body mass index (BMI) of <25 kg/m^2^ and normal electrocardiography (ECG) findings for AD, vs. a diastolic blood pressure (DBP) of <60 mmHg for ADH. As factors reducing exercise effectiveness, a hemoglobin of <12.4 g/dl for AD, vs. a history of arrhythmia and a VT percent max VO_2_ of <0.7 for ADH were identified.

Since the identification of the medication group as a factor significantly influencing the effect of exercise training on AD was completely unexpected, patient characteristics were analyzed for each medication subgroup. There were no distinct differences in patient characteristics between the medication subgroups (Table 1). The distribution of model-predicted individual risks (sum of log partial hazards) calculated from the main effects excluding exercise and medication at baseline also did not explain the differences in exercise effects by medication group (Supplementary Figure S2).

**Table 1:** Patient characteristics by baseline RASI and BB medication Status.

Characteristics	Medication at baseline				*P*-value*
	RASI + BB	RASI alone	BB alone	Neither	
	*N* = 1,888 (89.4%)	*N* = 107 (5.1%)	*N* = 107 (5.1%)	*N* = 10 (0.47%)	
Intervention assignment					
Usual care	946 (49.7)	53 (49.1)	67 (61.5)	4 (40.0)	0.056
Exercise	957 (50.3)	55 (50.9)	42 (38.5)	6 (60.0)	
Demographics					
Age, year	58 (50–67)	64 (55–73)	62 (50–72)	62 (57–64)	<0.001
Male sex	1,362 (71.6)	82 (75.9)	81 (74.3)	6 (60.0)	0.527
Vital signs					
Body mass index, kg/m^2^	30 (26–35)	28 (25–32)	29 (25–34)	27 (24–29)	0.116
Heart rate, bpm	70 (62–76)	72 (66–80)	72 (66–80)	80 (68–84)	<0.001
Systolic blood pressure, mmHg	110 (100–125)	112 (102–128)	112 (102–130)	110 (102–122)	0.261
Diastolic blood pressure, mmHg	70 (60–78)	70 (62–80)	70 (62–78)	70 (64–72)	0.831
Pulse pressure, mmHg	42 (34–50)	42 (34–55)	42 (34–58)	36 (32–43)	0.377
Left ventricle ejection fraction, %	25 (20–30)	24 (19–29)	23 (19–30)	21 (17–25)	0.133
NYHA class					
II	1,226 (64.4)	59 (54.6)	61 (56.0)	5 (50.0)	<0.05
III	661 (34.7)	48 (44.4)	45 (41.3)	5 (50.0)	0.054
IV	16 (0.8)	1 (0.9)	3 (2.8)	0 (0.0)	0.133
Beck score at baseline	8 (4–15)	9 (4–15)	9 (5–16)	14 (8–19)	0.641
Medical history					
Ischemic heart failure	967 (50.8)	59 (54.6)	62 (56.9)	8 (80.0)	0.365
Cardiac procedures					
Pacemaker	324 (17.0)	24 (22.2)	32 (29.4)	5 (50.0)	<0.01
Biventricular pacemaker	345 (18.1)	22 (20.4)	27 (24.8)	2 (20.0)	0.197
Automatic implantable cardioverter defibrillator	757 (39.8)	44 (40.7)	59 (54.1)	5 (50.0)	<0.05
Arrhythmias	748 (39.3)	65 (60.2)	59 (54.1)	8 (80.0)	<0.001
Hypertension	1,128 (59.3)	64 (59.3)	66 (60.6)	2 (20.0)	0.966
Stroke	184 (9.7)	16 (14.8)	15 (13.8)	3 (30.0)	0.099
Diabetes	600 (31.5)	34 (31.5)	44 (40.4)	0 (0.0)	0.156
COPD	186 (9.8)	29 (26.9)	17 (15.6)	0 (0.0)	<0.001
Laboratory tests					
Creatinine, mg/dl	1.2 (1.0–1.5)	1.3 (1.1–1.7)	1.3 (1.1–1.7)	0.9 (0.9–1.0)	<0.01
Total cholesterol, mg/dl	162 (137–191)	165 (138–188)	152 (125–189)	234 (182–250)	0.368
Hemoglobin A1c, percent	6.6 (5.9–7.8)	6.4 (6.0–7.4)	6.9 (6.4–8.2)	5.1 (5.1–5.1)	0.433
BNP, pg/ml	237 (104–502)	361 (162–694)	283 (140–949)	136 (103–260)	0.184
Hemoglobin, g/dl	14 (12–15)	14 (12–14)	13 (12–14)	14 (13–14)	0.278
Medications					
Loop diuretic	1,488 (78.2)	83 (76.9)	88 (80.7)	5 (50.0)	0.770
Digoxin	862 (45.3)	52 (48.1)	48 (44.0)	2 (20.0)	0.811
Nitrates	438 (23.0)	34 (31.5)	40 (36.7)	1 (10.0)	<0.001
6-min walk test					
Total distance walked, meter	375 (301–437)	347 (276–439)	329 (251–410)	366 (322–405)	<0.01
Kansas City cardiomyopathy questionnaire (KCCQ)					
KCCQ overall summary score	68 (52–83)	64 (45–82)	67 (48–81)	65 (48–76)	0.098
Cardiopulmonary exercise testing					
Exercise duration, min	9.8 (7.0–12.0)	8.8 (6.0–11.1)	8.5 (5.3–11.2)	11 (10–15)	<0.01
Peak VO_2_, ml/kg/min	14 (12–18)	14 (11–18)	13 (11–16)	18 (17–22)	<0.05
VO_2_ at ventilatory threshold, ml/kg/min	11 (9–12)	10 (9–13)	10 (8–12)	12 (11–13)	0.381
Ventilatory threshold percent max VO_2_	0.7 (0.6–0.8)	0.7 (0.6–0.8)	0.7 (0.7–0.8)	0.7 (0.6–0.7)	<0.05

Data are shown as *n* (%) for categorical variables and median (inter-quantile range) for continuous variables. Heterogeneity between groups were tested using ANOVA or the Kruskal–Wallis test for continuous variables and the chi-square test for categorical variables.

BB, beta-blocker; KCCQ, Kansas City cardiomyopathy questionnaire; NYHA, New York Heart Association; RASI, renin-angiotensin system inhibitor (i.e., angiotensin-converting enzyme inhibitor and/or angiotensin II receptor blocker); VO_2_, oxygen uptake.

* Comparing RASI + BB, RASI alone, and BB alone.

The parameter estimates for the final models for AD and ADH were shown in Supplementary Tables S2, S3, respectively. Forest plots for the main effects in the models for AD and ADH were shown in Supplementary Figures S3, S4, respectively. To understand the results of the Cox analysis more intuitively, the cumulative incidence curves for AD and ADH according to the baseline medication group are shown in Supplementary Figures S5, S6, respectively.

### 3.3 Score for predicting exercise eligibility

**Figure 4:**
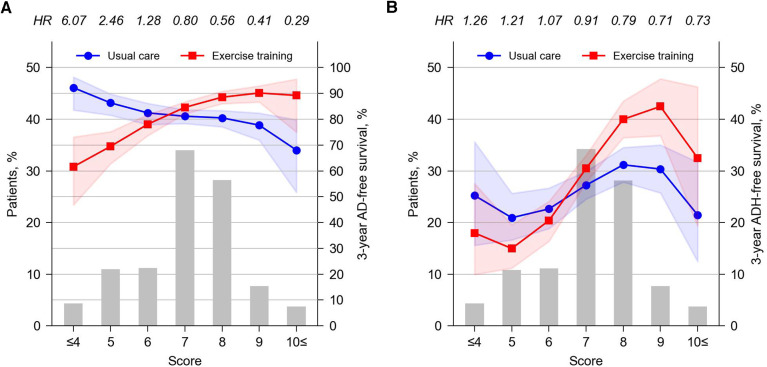
Patient distribution by score (left axis) and 3-year AD-free (**A**) or ADH-free (**B**) survival rates by intervention at each score (right axis). The survival rates per score and intervention were calculated using a Cox model accounting for predicted score (continuous variable) and intervention and their interaction, developed separately from the main analysis using Python's lifeline package (version 0.27.7). The values above the top axis indicate the HR for AD or ADH at each score (exercise training vs. usual care). Shared areas around the lines indicates the 95% CI. CI, confidence interval; HR, hazard ratio.

We developed a hypothetical clinical score, which estimates a patient's eligibility for exercise training according to a total of six predictors: treated with BBs (+3), pulse pressure ≥30 mmHg (+2), hemoglobin ≥12.4 g/dl (+2), history of stroke (+2), normal ECG findings (+1), and BMI <25 kg/m^2^ (+1) (Table 2). The score considered all the interaction effects identified in the final Cox model for AD, and for ease of understanding, all variables were assigned integer points by scaling the β coefficients while maintaining the gradient of the effects. Figures 4A,B show the distribution of patients by score (histogram), as well as the 3-year AD- or ADH-free survival rate, respectively, for patients belonging to each score who received usual care (lines with circles) or exercise training (lines with squares).

**Table 2:** Hypothetical Predictive Score for Exercise Eligibility in Patients with Chronic Heart Failure.

Variable	Points
Treated with β-blockers	+3
Pulse pressure ≥30 mmHg	+2
Hemoglobin ≥12.4 g/dl	+2
History of stroke	+2
Normal ECG	+1
Body mass index <25 kg/m^2^	+1
	Total score range 0–11

Score analysis showed that exercise training was clearly beneficial for AD in 39.6% of the total study population (i.e., patients with a score of 8 or higher) and in 45.3% of patients (i.e., those with scores of 6 or 7), exercise had no clear benefit on AD. Exercise training was estimated to increase AD in 15.3% of patients (i.e., patients with a score of 5 or lower) (Figure 4A). Note that although we used the results for AD to develop the presented score, an independent score developed from the results for ADH was also considered in a preliminary analysis (Supplementary Figure S7). When ADH score was used, again, exercise was predicted to be effective in reducing ADH only in approximately 40% of the study population (i.e., patients with a score of 4 or higher; Supplementary Figure S7C). However, such a subpopulation did not present a statistically significant exercise effect on AD (Supplementary Figure S7B), which was inferior to the results from the AD score, where patients who were predicted to benefit from exercise training in terms of AD also benefited in terms of ADH (Figures 4A,B). Similar discrepancies are also found in the subgroup for which exercise is predicted to be not beneficial by AD scores (i.e., the bottom 15% of AD scores), but the impact of such discrepancies would be less than sacrificing the discrimination of the top 40% of scores. Thus, the AD score was considered more discriminative and of greater clinical benefit for both AD and ADH.

## 4 Discussion

### 4.1 Main findings

To the best of our knowledge, this is the first analysis to comprehensively evaluate the effects of various factors on the outcome of exercise training in patients with chronic heart failure. The HF-ACTION trial demonstrated that compared to the usual care group, exercise training did not statistically affect the rate of AD but did reduce the rate of ADH (9). On the other hand, the present analysis suggested that the effects of exercise training may not necessarily apply uniformly to all patients with chronic heart failure. In this analysis, we paid best efforts to avoid detecting meaningless covariates by considering correlation between the covariates and applying the Boruta method. However, Boruta is a heuristic; there are no strict guarantees about its output although it is an interesting statistical tool. In addition, this analysis is *post-hoc* in design and the risk of confounding should always be considered. Even though, this study is expected to provide useful information that may contribute to the individualization of exercise therapy for patients of chronic heart failure.

### 4.2 Exercise effect and taking BBs

In patients with chronic heart failure, exercise training improves functional capacity, increases muscle strength, and improves quality of life (1–5, 23–25). However, exercise could excessively increase cardiac workload by increasing heart rate via sympathetic nervous system activation (14, 26) and may also induce arrhythmia when the intensity is increased (27). This study suggested that the effect of exercise training on ADH was larger in patients taking BBs at baseline, which may have occurred because BBs suppressed excessive sympathetic nervous system activation by exercise training and reduced the increased demand for myocardial energy due to increased heart rate (16). BBs may also have prevented sudden death by suppressing the onset of ventricular arrhythmias (28–30). A previous model-based meta-analysis showed that cardiac load reduction by BB was associated with improved prognosis of patients with chronic heart failure (13). Taken together, the present analysis indicated that BBs may play an important role in maximizing benefits of exercise training and improving tolerance.

### 4.3 Exercise effect and patient backgrounds

In addition to medication, the study identified several factors that could influence the effects of exercise training. In particular, pulse pressure, the difference between systolic and DBP, was identified as one of the most significant factors in both analyses for AD and ADH, with lower values being associated with adverse exercise effects. Similarly, low hemoglobin levels were identified as a factor negatively affecting exercise effectiveness. It is known that in patients with advanced chronic heart failure with reduced cardiac contractility, pulse pressure decreases due to a reduced systolic blood pressure (31). It has also been reported that patients with chronic heart failure complicated by anemia have a reduced oxygen supply to the heart and a poorer prognosis compared to patients without anemia (32–36). Given these pathological changes, our results likely reflect an increased risk of death and hospitalization in these patients due to the cardiac overload caused by exercise.

Other important factors identified as affecting exercise effects included abnormal ECG findings/arrhythmia and BMI. Our results suggest that exercise training is not recommended in patients with abnormal ECG findings or arrhythmia, which seems reasonable considering the risk of sudden death (27). With regard to BMI, the risk of AD was decreased by exercise training in patients with a BMI of <25 kg/m^2^, suggesting that maintaining one's muscle strength through exercise may contribute to the prevention of cachexia or inhibition of its progression (37–40). The present results overall are consistent with general knowledge on the pathogenesis of chronic heart failure. A history of stroke was a positive effect on exercise but it was also a factor that increased the risk of AD. This result is difficult to explain currently but it might be a confounding of use of antithrombotic drugs, because patients with a history of stroke were more likely to be prescribed antithrombotic drugs (41–44).

### 4.4 Factors influencing outcomes

In addition to the interactions with exercise, many prognostic factors have been reported for chronic heart failure. O'Connor et al. reported that exercise duration in the CPX test, BUN, and sex were important for AD and ADH based on the analysis of patient information of the HF-ACTION trial (45). In both studies, exercise capacity, renal function, and sex were selected. Overall, although this study is novel in that it includes multiple interactions with exercise, the analysis of other prognostic factors was considered consistent with that of the previous studies.

### 4.5 Exercise effect and taking RASIs and other medications

The present analysis suggested that exercise is not beneficial in patients taking only RASIs. In fact, the usual care group without exercise training taking only RASIs was associated with the lowest risk for AD in the study. This trend was consistent for ACEIs and ARBs that comprise RASI; no deaths occurred in subjects taking ARB alone in the absence of exercise training (Supplementary Table S4). Since RASI could alter serum potassium by affecting aldosterone secretion, it is not impossible that this could have caused some systematic change in the risk of events in chronic heart failure patients (7, 46). However, it is inappropriate to judge the superiority or inferiority of medications rather than their effect on exercise training based on the results of the present analysis. The HF-ACTION trial was randomized for exercise but not for medications, and only a small number of patients (5.1% of the total) took RASI alone at baseline. In fact, the current chronic heart failure guidelines recommend the combined use of RASIs and BBs as first-line therapy based on the solid evidences (7, 46).

Since the HF-ACTION trial was conducted in 2003–2008, the medication algorithms for chronic heart failure differed from the current ones. Sodium-glucose cotransporter-2 inhibitors and angiotensin receptor-neprilysin inhibitors (ARNIs) have since been demonstrated (47) to improve chronic heart failure prognosis (7, 46). Although the impact of these therapies on exercise training could not be evaluated in this study, given the similarity in the action between RASIs and ARNIs, similar caution might be required for patients who take ARNIs without BBs.

Regarding mineralocorticoid antagonists, spironolactone and eplerenone were prescribed at baseline in 42% and 3% of patients, respectively. Variables related to mineralocorticoids were included in the analysis but was not selected for the final model.

### 4.6 Exercise Recommendation based on the score

A hypothetical score was constructed based on the results of Cox proportional hazards analysis in terms of AD to categorize patients for whom exercise training is recommended or not recommended. The study may inform the extent to which prognosis changes when patients in each score do or do not perform exercise training, along with their uncertainty (Figure 3). Patients with a score of 8 or higher are considered important to ensure exercise training, whereas patients with intermediate scores of 6–7, the outcome may be affected little from the decision on exercise training. Exercise prescription may need to be carefully avoided for patients with scores of 5 or less. This analysis implied that 40% of the entire study population appeared to clearly benefit from exercise training, while the effect of exercise was unclear in 45% of patients and increased AD in the remaining 15% of patients. Given that exercise training is widely recommended in the current management of chronic heart failure (7), the proposed score would be clinically meaningful. However, these have not yet been validated by multiple studies. Therefore, their generalizability to chronic heart failure patients remains to be confirmed in the future.

### 4.7 Limitations

The population included in this analysis consisted of patients with stable chronic heart failure, a reduced left ventricular ejection fraction (≤35%), NYHA class II–IV despite treatment, and the ability to perform exercise. While we believe that the analysis in this study is appropriate for these patients, it may not be applicable to other populations, such as those using medications that were not approved at the time the HF-ACTION trial was conducted, those who had a recent cardiovascular event or comorbidities or limitations that could interfere with exercise training. In addition, the appropriateness of exercise training may vary greatly by exercise type and intensity. If exercise is anticipated to be not beneficial, future studies would be needed to examine the effects of reducing exercise intensity. Given the limitations involved in *post-hoc* analyses of a single clinical trial, new exercise trials in patients with HF would be necessary to be conducted to confirm the factors detected and the appropriateness of the score.

## 5 Conclusions

These results highlighted that the effects of exercise training may not necessarily apply uniformly to all patients with chronic heart failure. The choice of exercise training in patients with chronic heart failure requires careful judgment that considers patient condition and also possibly medications. Further validation of this study would be required in the future.

## Data Availability

The datasets presented in this article are not readily available because the data analyzed in this study was obtained from National Heart, Lung, and Blood Institute with determined procedure and permission of National Heart, Lung, and Blood Institute. Requests to access the datasets should be directed to https://biolincc.nhlbi.nih.gov/home/. Further enquiries can be directed to the corresponding author.
