# Supplementary material for: Exercise training outcomes in patients with chronic heart failure with reduced ejection fraction depend on patient background
Source: Front Cardiovasc Med. 2024 Jan 31;11:1330235. doi: 10.3389/fcvm.2024.1330235 (PMC10869166; doi:10.3389/fcvm.2024.1330235)
Supplement: Supplementary file 1 [file Datasheet1.docx]

Supplementary Material

# Supplementary Figures

**Supplementary Figure S1.** Importance (probability of selection, %) of main effects assessed using 1000 bootstrap datasets by the Boruta method. (A) AD, (B) ADH. FDR, false discovery rate.

**Supplementary Figure S2.** Model-predicted individual risks (log partial hazard) calculated from the main effects (excluding exercise and medication at baseline) identified in the Cox analysis. P values were calculated by t-test.

**Supplementary Figure S3.** Forest plots of main effects included in the model for AD. The reference population for calculating HR vs Ref. is the usual care group in the overall study population.

**Supplementary Figure S4.** Forest plots of main effects included in the model for ADH. The reference population for calculating HR vs Ref. is the usual care group in the overall study population.


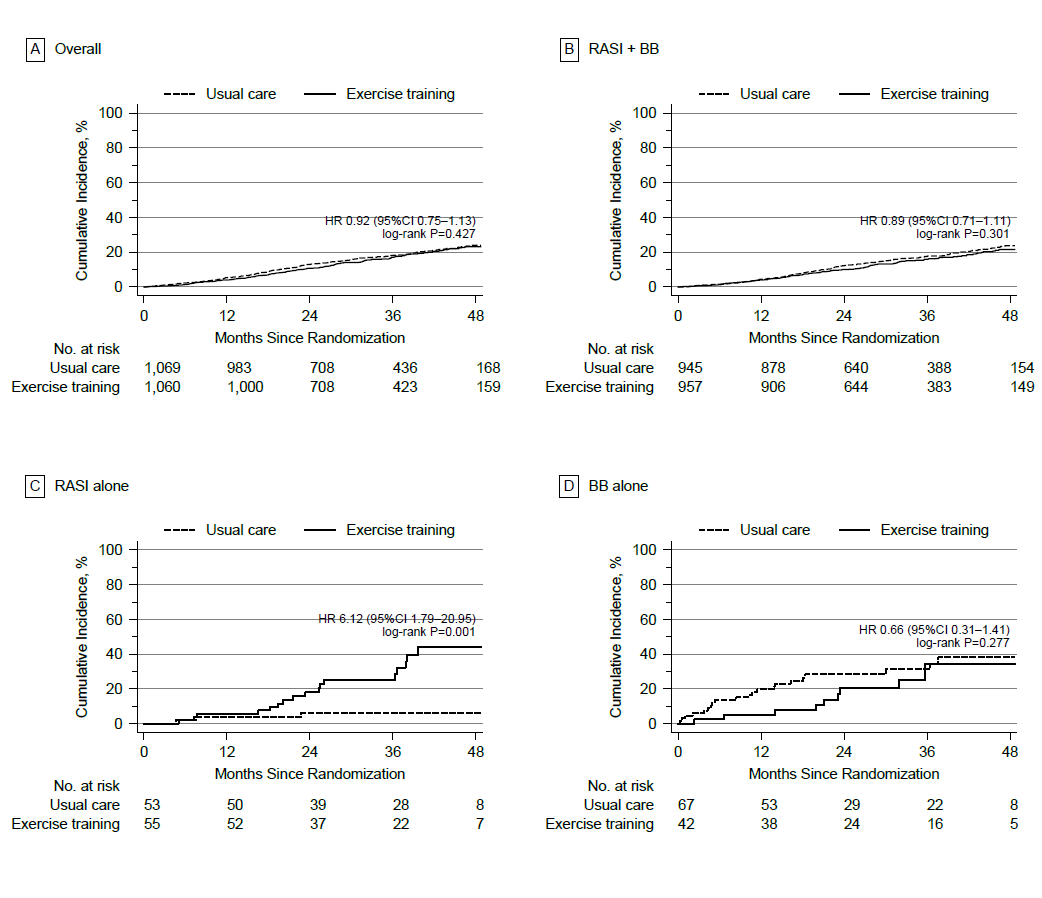


**Supplementary Figure S5.** Cumulative incidence of all-cause death by medication at baseline. The cumulative incidence of all-cause death in each medication group was directly compared. A Overall study population. B Patients taking both RASIs and BBs at baseline. C Patients taking RASIs alone at baseline. D Patients taking BBs alone at baseline. BB, beta-blocker; CI, confidence interval; HR, hazard ratio; RASI, renin-angiotensin system inhibitor.


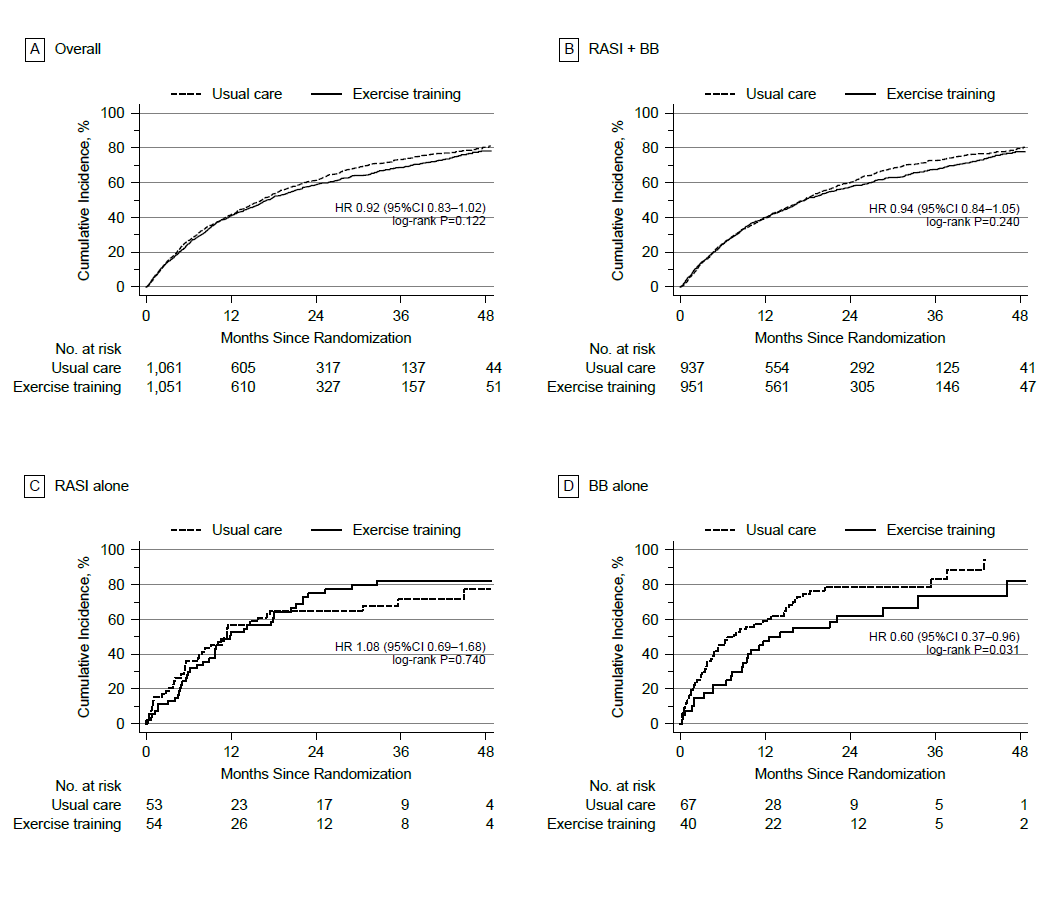


**Supplementary Figure S6.** Cumulative incidence of all-cause death or hospitalization by medication at baseline. The cumulative incidence of all-cause death or hospitalization for each medication group was directly compared. A Overall study population. B Patients taking both RASIs and BBs at baseline. C Patients taking RASIs alone at baseline. D Patients taking BBs alone at baseline. BB, beta-blocker; CI, confidence interval; HR, hazard ratio; RASI, renin-angiotensin system inhibitor.


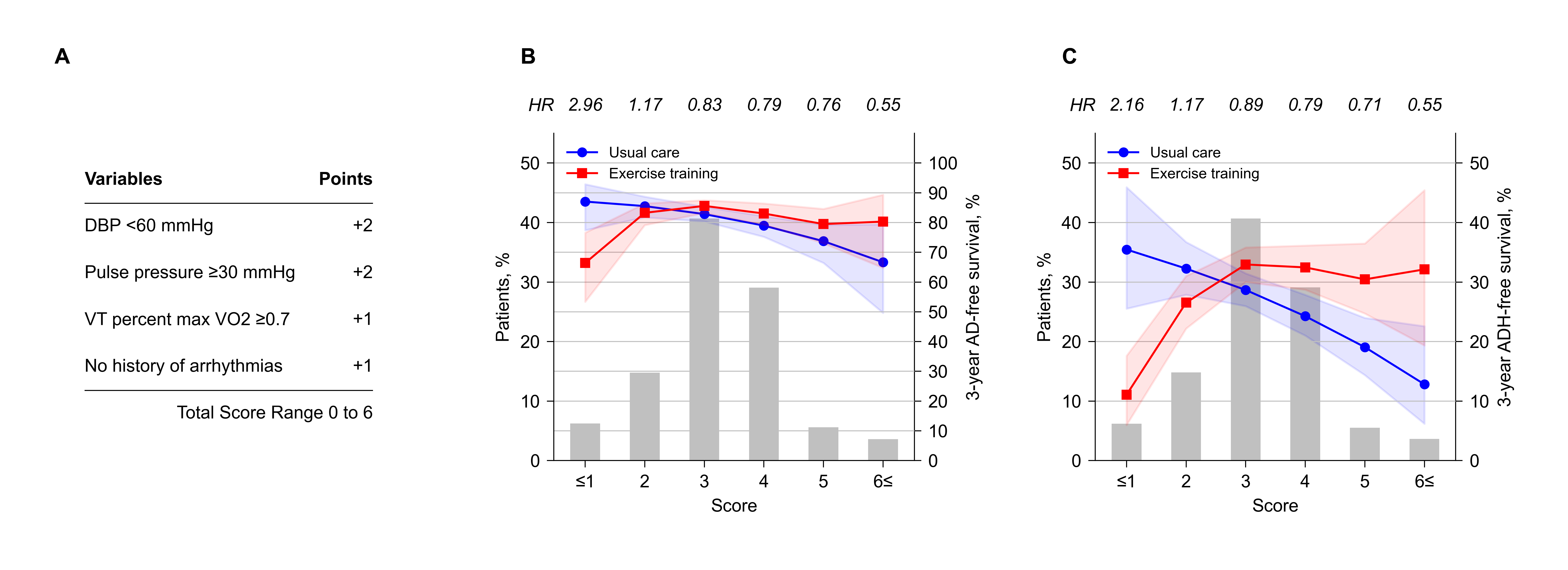


**Supplementary Figure S7.** Hypothetical predictive score derived from the Cox model for ADH.

# Supplementary Tables

**Supplementary Table S1.** List of variables used as candidate covariates in Cox regression analysis

| **Domain** | **No** | **Variable** | **Label** | **Type** | **Cutoff** | **Unit** | **Missing (%)** | **Excluded** |
| --- | --- | --- | --- | --- | --- | --- | --- | --- |
| **Demographics** | **1** | **age** | Age | continuous | 50;60;70 | years | 0 |  |
|  | **2** | **male** | Mele sex | categorical |  |  | 0 |  |
|  | **3** | **white** | Race, White | categorical |  |  | 1.5 |  |
|  | **–** | **black** | Race, Black | categorical |  |  | 1.5 | Correlated with white (chi square >300) |
|  | **4** | **asian** | Race, Asian, Amer Ind, Pac. Isl | categorical |  |  | 1.5 |  |
| **Vital Sign** | **5** | **bmi** | Body mass index | continuous | 25;30;35 | kg/m^2 | 0.2 |  |
|  | **–** | **weightkg** | Body weight | continuous | 75;90;110 | kg | 0 | Correlated with bmi (spearman >0.8) |
|  | **6** | **basehr** | Heart rate | continuous | 60;70;80 | bpm | 0.2 |  |
|  | **7** | **pulsepr** | Pulse pressure | continuous | 30;40;50 | mmHg | 0.2 |  |
|  | **8** | **sbp** | Systolic blood pressure | continuous | 100;110;125 | mmHg | 0.2 |  |
|  | **9** | **dbp** | Diastolic blood pressure | continuous | 60;70;80 | mmHg | 0.2 |  |
|  | **–** | **egfr** | eGFR | continuous | 50;65;80 | mL/min/1.73 m^2 | 10.2 | Correlated with creatnc (spearman >0.8) |
| **Subject Status** | **10** | **bestlvef** | Best available LVEF | continuous | 20;25;30 | % | 0.2 |  |
|  | **11** | **nyhacl** | NHYA class | continuous | 3;4 |  | 0 |  |
|  | **12** | **beckb** | Beck score | continuous | 4;8;15 |  | 0.4 |  |
|  | **13** | **ccsangcl** | Current CCS angina class | continuous | 1;2 |  | 0.1 |  |
|  | **14** | **hfhosp** | No. of HF hospitalizations in last 6 months | continuous | 1;2;3 |  | 0.8 |  |
|  | **15** | **hospsxmo** | No. of hospitalizations in last 6 months | continuous | 1;2;3 |  | 0.7 |  |
|  | **–** | **ischemic** | Ischemic etiology | categorical |  |  | 0 | Correlated with llwa (chi square >300) |
|  | **16** | **renaldys** | Renal dysfunction | categorical |  |  | 10.2 |  |
|  | **17** | **smokenever** | Never smoker | categorical |  |  | 0.5 |  |
|  | **18** | **smokecurr** | Current smoker | categorical |  |  | 0.5 |  |
|  | **–** | **alcohol** | Alcohol use | categorical |  |  | 1.8 | Correlated with nodrink (chi square >300) |
|  | **19** | **nodrink** | Patient no longer drinks | categorical |  |  | 0 |  |
| **ECG** | **20** | **ecgcond3** | Normal ECG | categorical |  |  | 2.5 |  |
| **CPX** | **21** | **cpxdur** | Exercise duration in CPX test | continuous | 7;10;12 | minutes | 0.9 |  |
|  | **22** | **peakvo2** | Peak VO2 | continuous | 11;14;17 | mL/kg/min | 2.4 |  |
|  | **23** | **vevco2** | Ve/VCO2 slope | continuous | 24;30;34 |  | 3.2 |  |
|  | **24** | **peakrer** | Peak respiratory exchange ratio | continuous | 1.0;1.1;1.2 |  | 3.1 |  |
|  | **–** | **vo2atvt** | VO2 at ventilatory threshold | continuous | 8.8;10.6;12.5 | mL/min/kg | 16.8 | Correlated with peakvo2 (spearman >0.8) |
| **CPX Test** | **25** | **cpxrsthr** | Resting heart rate | continuous | 60;70;80 | bpm | 0.1 |  |
|  | **26** | **hrpeakex** | HR at peak exercise | continuous | 100;120;130 | bpm | 0.1 |  |
|  | **–** | **cpxhrr** | Heart rate reserve | continuous | 35;50;60 | bpm | 0.1 | Correlated with hrpeakex (spearman >0.8) |
|  | **27** | **peakrpe** | Borg RPE score at peak exercise | continuous | 15;17;19 |  | 0.5 |  |
|  | **28** | **vtpctvo2** | VT percent max VO2 | continuous | 0.6;0.7;0.8 |  | 16.8 |  |
|  | **29** | **pko2puls** | Peak oxygen pulse | continuous | 0.10;0.12;0.15 | mL/kg | 2.4 |  |
|  | **–** | **tmrerone** | Time to RER = 1.0 | continuous | 6;8;10 | minutes | 22.7 | Correlated with cpxdur (spearman >0.8) |
|  | **30** | **hrwl2** | Heart rate at the end of WL 2 | continuous | 80;90;100 | bpm | 5.3 |  |
|  | **–** | **ouesall** | Oxygen uptake efficiency slope measured from entire CPX test | continuous | 1200;1600;2000 | mL/min/log(L/min) | 2.6 | Correlated with abspkvo2 (spearman >0.8) |
|  | **–** | **ouesupto6** | Oxygen uptake efficiency slope measured from the first 6 minutes of CPX test | continuous | 1200;1600;2000 | mL/min/log(L/min) | 18.5 | Correlated with ouesall (spearman >0.8) |
|  | **–** | **ouesuptorer1** | Oxygen uptake efficiency slope measured during CPX test while RER = 1.0 | continuous | 1200;1600;2000 | mL/min/log(L/min) | 27 | Correlated with ouesall (spearman >0.8) |
|  | **–** | **weber** | Weber class | continuous | 2;3;4 |  | 2.4 | Correlated with peakvo2 (spearman >0.8) |
|  | **31** | **abspkvo2** | Absolute peak VO2 | continuous | 1000;1300;1700 | mL/min | 2.5 |  |
| **6-Min Walk Test** | **32** | **sixmwlka** | Six-minute walk: able to walk?, Yes | categorical |  |  | 0 |  |
|  | **33** | **sixmwlks** | Six minute walk: symptomatic?, Yes | categorical |  |  | 3.8 |  |
|  | **34** | **sixmwlkd** | Six-minute walk distance, subjects able to walk | continuous | 300;370;440 | meters | 2.2 |  |
|  | **35** | **walkwork** | Work done during 6MW, subjects able to walk | continuous | 240;320;400 | kJ | 2.2 |  |
| **Echocardiography** | **–** | **eprimevel** | E prime velocity | continuous |  |  | 60.6 | Missing in >30% of samples |
|  | **–** | **eeprimevelrat** | E/E prime velocity ratio | continuous |  |  | 65.4 | Missing in >30% of samples |
|  | **–** | **ewavsept** | Tissue Doppler E wave (septal annulus) | continuous |  |  | 51.1 | Missing in >30% of samples |
|  | **–** | **ewavlat** | Tissue Doppler E wave (lateral annulus) | continuous |  |  | 50 | Missing in >30% of samples |
|  | **–** | **mvpkevel** | Doppler MV peak E velocity | continuous |  |  | 33.2 | Missing in >30% of samples |
|  | **–** | **lveddt** | Left ventricular early diastolic deceleration time | continuous |  |  | 30.8 | Missing in >30% of samples |
|  | **–** | **lftatrdm** | Left atrial dimension | continuous | 4.0;4.5;5.0 | cm | 29.2 |  |
|  | **37** | **lftvmass** | Left ventricular mass | continuous | 200;300;400 | g | 29.3 |  |
|  | **38** | **mitregrg** | Mitral regurgitation | continuous | 10;20;30 | % | 22.3 |  |
|  | **39** | **lftvdiad** | Left ventricular diastolic dimension | continuous | 5.9;6.6;7.4 | cm | 29.4 |  |
|  | **–** | **eavelrat** | MV peak E velocity / A velocity | continuous |  |  | 33.1 | Missing in >30% of samples |
|  | **–** | **mvpkevlv** | MV peak E velocity - Valsalva | continuous |  |  | 75.1 | Missing in >30% of samples |
|  | **–** | **mvpkavlv** | MV peak A velocity - Valsalva | continuous |  |  | 75.1 | Missing in >30% of samples |
|  | **–** | **mvearatv** | MV E/A ratio - Valsalva | continuous |  |  | 75 | Missing in >30% of samples |
| **Laboratory Value** | **40** | **creatnc** | Creatinine | continuous | 1.0;1.2;1.5 | mg/dL | 10.2 |  |
|  | **41** | **bun** | BUN | continuous | 15;20;30 | mg/dL | 13 |  |
|  | **–** | **totcholc** | Total cholesterol | continuous |  | mg/dL | 35.4 | Missing in >30% of samples |
|  | **–** | **hdl** | High-density lipoprotein | continuous |  | mg/dL | 38.5 | Missing in >30% of samples |
|  | **–** | **ldl** | Low-density lipoprotein | continuous |  | mg/dL | 39.9 | Missing in >30% of samples |
|  | **–** | **hema1cc** | Hemoglobin A1c | continuous |  | % | 73.4 | Missing in >30% of samples |
|  | **–** | **bnp** | BNP | continuous |  | pg/mL | 64.2 | Missing in >30% of samples |
|  | **–** | **probnp** | Pro-BNP | continuous |  | pg/mL | 96.5 | Missing in >30% of samples |
|  | **42** | **hemogloc** | Hemoglobin | continuous | 12.4;13.5;14.6 | g/dL | 24.1 |  |
| **Medical History** | **43** | **valvsurg** | History of prior valve surgery | categorical |  |  | 0 |  |
|  | **44** | **revasc** | History of prior revascularization | categorical |  |  | 0 |  |
|  | **–** | **priorpci** | History of prior PCI | categorical |  |  | 0 | Correlated with ischemic (chi square >300) |
|  | **–** | **cabg** | History of prior CABG | categorical |  |  | 0 | Correlated with ischemic (chi square >300) |
|  | **45** | **copd** | History of COPD | categorical |  |  | 0.9 |  |
|  | **–** | **diabetes** | History of diabetes | categorical |  |  | 0 | Correlated with insulin (chi square >300) |
|  | **–** | **depress** | History of depression | categorical |  |  | 0 | Correlated with ssri (chi square >300) |
|  | **46** | **angina** | History of angina | categorical |  |  | 0 |  |
|  | **–** | **priormi** | History of myocardial infarction | categorical |  |  | 0 | Correlated with ischemic (chi square >300) |
|  | **–** | **pvd** | History of peripheral vascular disease | categorical |  |  | 0.5 | Correlated with claud (chi square >300) |
|  | **47** | **stroke** | History of stroke | categorical |  |  | 0 |  |
|  | **48** | **hyperten** | History of hypertension | categorical |  |  | 0.6 |  |
|  | **–** | **arr** | History of arrhythmias | categorical |  |  | 0 | Correlated with vtach (chi square >300) |
|  | **–** | **afibflut** | History of atrial fibrillation or flutter | categorical |  |  | 0 | Correlated with arr (chi square >300) |
|  | **49** | **brady** | History of symptomatic bradycardia | categorical |  |  | 0 |  |
|  | **50** | **vtach** | History of sustained ventricular tachycardia/ventri | categorical |  |  | 0 |  |
|  | **–** | **hlipid** | History of hyperlipidemia | categorical |  |  | 0.1 | Correlated with llwa (chi square >300) |
|  | **51** | **claud** | History of claudication | categorical |  |  | 0 |  |
|  | **52** | **cancer** | History of cancer in last 5 years | categorical |  |  | 0.6 |  |
|  | **–** | **aicd** | On an automatic implantable cardioverter-defibrillator at baseline | categorical |  |  | 0 | Correlated with vtach (chi square >300) |
|  | **53** | **bivpacer** | On a bi-ventricular pacemaker at baseline | categorical |  |  | 0 |  |
|  | **54** | **pacer** | On a pacemaker at baseline | categorical |  |  | 0 |  |
| **Medication** | **55** | **loopdiur** | Use of loop diuretic at baseline | categorical |  |  | 0 |  |
|  | **56** | **nonloop** | Use of non-loop diuretic (excluding aldosterone antagonist) at baseline | categorical |  |  | 0 |  |
|  | **–** | **hmgcoari** | Use of HMG-CoA reductase inhibitor at baseline | categorical |  |  | 0 | Correlated with llwa (chi square >300) |
|  | **57** | **digoxin** | Use of digoxin at baseline | categorical |  |  | 0 |  |
|  | **58** | **nitrate** | Use of nitrates at baseline | categorical |  |  | 0 |  |
|  | **59** | **calcchbl** | Use of calcium channel blocker at baseline | categorical |  |  | 0 |  |
|  | **60** | **insulin** | Use of insulin at baseline | categorical |  |  | 0 |  |
|  | **–** | **spiro** | Use of spironolactone at baseline | categorical |  |  | 0 | Correlated with aldant (chi square >300) |
|  | **61** | **epleren** | Use of eplerenone at baseline | categorical |  |  | 0 |  |
|  | **62** | **aldant** | Use of aldosterone antagonist (spiro. or epleren.) at baseline | categorical |  |  | 0 |  |
|  | **63** | **aspirin** | Use of aspirin at baseline | categorical |  |  | 0 |  |
|  | **64** | **llwa** | Use of liquid-lowering agent at baseline | categorical |  |  | 0 |  |
|  | **65** | **arrhyt** | Use of antiarrhythmic at baseline | categorical |  |  | 0 |  |
|  | **66** | **ssri** | Use of SSRI at baseline | categorical |  |  | 0 |  |
| **Questionnaire** | **–** | **kccqosb** | KCCQ overall summary score at baseline | continuous | 50;65;80 |  | 0 | Correlated with kccqslsb (spearman >0.8) |
|  | **–** | **kccqcsb** | KCCQ clinical summary score at baseline | continuous | 60;75;90 |  | 0 | Correlated with kccqplb (spearman >0.8) |
|  | **67** | **kccqplb** | KCCQ physical limitation score at baseline | continuous | 50;70;90 |  | 0.8 |  |
|  | **68** | **kccqslsb** | KCCQ social limitation score at baseline | continuous | 40;60;90 |  | 1.7 |  |
|  | **–** | **kccqtsb** | KCCQ total symptom score at baseline | continuous | 60;75;90 |  | 0 | Correlated with kccqsfb (spearman >0.8) |
|  | **69** | **kccqssb** | KCCQ symptom stability score at baseline | continuous | 25;50;75 |  | 0.6 |  |
|  | **70** | **kccqsfb** | KCCQ symptom frequency score at baseline | continuous | 60;75;90 |  | 0.1 |  |
|  | **–** | **kccqsbb** | KCCQ symptom burden score at baseline | continuous | 60;75;90 |  | 0 | Correlated with kccqsfb (spearman >0.8) |
|  | **71** | **kccqseb** | KCCQ self-efficacy score at baseline | continuous | 70;90;100 |  | 0.1 |  |
|  | **72** | **kccqqolb** | KCCQ quality of life score at baseline | continuous | 40;60;80 |  | 0.1 |  |
|  | **73** | **eurother** | EuroQol thermometer response | continuous | 50;70;80 |  | 2.5 |  |

**Supplementary Table S2.** Results of Cox regression analysis for AD

| **Effects** | **Label** | **Coefficient** | **95%CI** |
| --- | --- | --- | --- |
| **Effects of exercise by medication at baseline** |  |  |  |
| exercise__rasi1_beta1 | Exercise effect in patients treated with both RASI and BB at baseline | -0.15 | -0.27, -0.04 |
| exercise__rasi1_beta0 | Exercise effect in patients treated with RASI but not BB at baseline | 1.58 | 1.18, 1.94 |
| exercise__rasi0_beta1 | Exercise effect in patients treated with BB but not RASI at baseline | -0.54 | -1.07, -0.09 |
| exercise__rasi0_beta0 | Exercise effect in patients treated with neither RASI nor BB at baseline | 0 fixed | – |
| **Interactions with exercise training** |  |  |  |
| exercise * bmi_lt_25 | Interaction effect between exercise and bmi_lt_25 | -0.55 | -1.04, -0.07 |
| exercise * pulsepr_lt_30 | Interaction effect between exercise and pulsepr_lt_30 | 0.86 | 0.20, 1.53 |
| exercise * ecgcond3_eq_1 | Interaction effect between exercise and ecgcond3_eq_1 | -0.51 | -1.00, -0.07 |
| exercise * hemogloc_lt_12p4 | Interaction effect between exercise and hemogloc_lt_12p4 | 0.93 | 0.46, 1.49 |
| exercise * stroke_eq_1 | Interaction effect between exercise and stroke_eq_1 | -1.15 | -1.94, -0.37 |
| **Main effects** |  |  |  |
| **Medication at baseline** |  |  |  |
| acearb1_betab1 | Treated with both RASI and BB at baseline | 0 (Ref.) | – |
| acearb1_betab0 | Treated with RASI but not BB at baseline | -1.15 | -1.39, -0.86 |
| acearb0_betab1 | Treated with BB but not RASI at baseline | 0.70 | 0.44, 1.00 |
| acearb0_betab0 | Treated with neither RASI nor BB at baseline | 0.80 | 0.40, 1.18 |
| **Included in interactions with exercise training** |  |  |  |
| bmi_lt_25 | Body mass index <25 kg/m^2 | 0.59 | 0.28, 0.92 |
| pulsepr_lt_30 | Pulse pressure <30 mmHg | -0.19 | -0.68, 0.26 |
| ecgcond3_eq_1 | Normal ECG, Yes | 0.13 | -0.19, 0.45 |
| hemogloc_lt_12p4 | Hemoglobin <12.4 g/dL | -0.31 | -0.74, 0.08 |
| stroke_eq_1 | History of stroke, Yes | 0.41 | -0.06, 0.80 |
| **Others** |  |  |  |
| male_eq_0 | Female sex | -0.41 | -0.70, -0.17 |
| egfr_lt_65 | eGFR <65 mL/min/1.73 m^2 | 0.57 | 0.36, 0.85 |
| vevco2_ge_34 | Ve/VCO2 slope ≥34 | 0.58 | 0.34, 0.81 |
| hrpeakex_lt_100 | HR at peak exercise <100 bpm | 0.67 | 0.42, 0.90 |
| valvsurg_eq_1 | History of prior valve surgery, Yes | 0.52 | 0.13, 0.85 |

**Supplementary Table S3.** Results of Cox regression analysis for all-cause death or all-cause hospitalization

| **Effects** | **Label** | **Coefficient** | **95%CI** |
| --- | --- | --- | --- |
| **Effects of exercise by medication at baseline** |  |  |  |
| exercise__rasi1_beta1 | Exercise effect in patients treated with both RASI and BB at baseline | -0.12 | -0.23, -0.00 |
| exercise__rasi1_beta0 | Exercise effect in patients treated with RASI but not BB at baseline | -0.04 | -0.52, 0.42 |
| exercise__rasi0_beta1 | Exercise effect in patients treated with BB but not RASI at baseline | -0.57 | -1.03, -0.10 |
| exercise__rasi0_beta0 | Exercise effect in patients treated with neither RASI nor BB at baseline | 0 fixed | – |
| **Interactions with exercise training** |  |  |  |
| exercise * pulsepr_lt_30 | Interaction effect between exercise and pulsepr_lt_30 | 0.70 | 0.39, 1.03 |
| exercise * dbp_lt_60 | Interaction effect between exercise and dbp_lt_60 | -0.48 | -0.79, -0.20 |
| exercise * vtpctvo2_lt_0p7 | Interaction effect between exercise and vtpctvo2_lt_0p7 | 0.31 | 0.07, 0.55 |
| exercise * stroke_eq_1 | Interaction effect between exercise and stroke_eq_1 | -0.44 | -0.78, -0.12 |
| exercise * arr_eq_1 | Interaction effect between exercise and arr_eq_1 | 0.45 | 0.23, 0.67 |
| **Main effects** |  |  |  |
| **Medication at baseline** |  |  |  |
| acearb1_betab1 | Treated with both RASI and BB at baseline | 0 (Ref.) | – |
| acearb1_betab0 | Treated with RASI but not BB at baseline | 0.00 | -0.37, 0.34 |
| acearb0_betab1 | Treated with BB but not RASI at baseline | 0.40 | 0.11, 0.70 |
| acearb0_betab0 | Treated with neither RASI nor BB at baseline | 0.66 | -0.09, 1.71 |
| **Included in interactions with exercise training** |  |  |  |
| pulsepr_lt_30 | Pulse pressure <30 mmHg | -0.20 | -0.44, 0.06 |
| dbp_lt_60 | Diastolic blood pressure <60 mmHg | 0.26 | 0.07, 0.48 |
| vtpctvo2_lt_0p7 | VT percent max VO2 <0.7 | -0.38 | -0.56, -0.20 |
| stroke_eq_1 | History of stroke, Yes | 0.31 | 0.11, 0.52 |
| arr_eq_1 | History of arrhythmias, Yes | 0.05 | -0.10, 0.21 |
| **Others** |  |  |  |
| white_eq_0 | Race, White, No | 0.18 | 0.05, 0.30 |
| pulsepr_lt_40 | Pulse pressure <40 mmHg | 0.21 | 0.06, 0.35 |
| sbp_lt_110 | Systolic blood pressure <110 mmHg | -0.23 | -0.39, -0.07 |
| dbp_lt_70 | Diastolic blood pressure <70 mmHg | 0.23 | 0.10, 0.36 |
| egfr_lt_50 | eGFR <50 mL/min/1.73 m^2 | 0.33 | 0.20, 0.45 |
| bestlvef_lt_20 | Best available LVEF <20 % | 0.22 | 0.09, 0.33 |
| ecgcond3_eq_1 | Normal ECG, Yes | -0.22 | -0.35, -0.10 |
| cpxdur_ge_12 | Exercise duration in CPX test ≥12 minutes | -0.28 | -0.43, -0.15 |
| pko2puls_lt_0p10 | Peak oxygen pulse <0.10 mL/kg | 0.26 | 0.13, 0.38 |
| sixmwlks_eq_1 | Six minute walk: symptomatic, Yes | 0.27 | 0.11, 0.43 |
| sixmwlkd_lt_370 | Six-minute walk distance <370 meters | 0.18 | 0.07, 0.30 |
| nitrate_eq_1 | Use of nitrates at baseline, Yes | 0.19 | 0.06, 0.31 |
| kccqosb_lt_50 | KCCQ overall summary score at baseline <50 | 0.29 | 0.16, 0.42 |
| kccqssb_lt_50 | KCCQ symptom stability score at baseline <50 | 0.49 | 0.30, 0.69 |

**Supplementary Table S4.** Clinical outcomes by baseline RASI and BB medication status

| Medication at baseline | | Study arm | N | Events in 4 years (%) | |
| --- | --- | --- | --- | --- | --- |
|  |  |  |  | AD | ADH |
| RASI + BB | | Usual care | 945 | 157 (16.6) | 639 (67.6) |
|  |  | Exercise | 957 | 141 (14.7) | 617 (64.5) |
| RASI alone | ACEI + ARB | Usual care | 53 | 3 (5.66) | 37 (69.8) |
|  |  | Exercise | 55 | 17 (30.9) | 42 (76.4) |
|  | ACEI | Usual care | 35 | 3 (8.57) | 24 (68.6) |
|  |  | Exercise | 41 | 15 (36.6) | 32 (78.1) |
|  | ARB | Usual care | 16 | 0 (0.00) | 12 (75.0) |
|  |  | Exercise | 12 | 2 (16.7) | 8 (66.7) |
| BB alone | | Usual care | 67 | 21 (31.3) | 54 (80.6) |
|  |  | Exercise | 42 | 10 (23.8) | 27 (64.3) |
| Neither | | Usual care | 4 | 2 (50.0) | 3 (75.0) |
|  |  | Exercise | 6 | 0 (0.00) | 5 (83.3) |

BB, beta-blocker; RASI, renin-angiotensin system inhibitor (i.e., angiotensin-converting enzyme inhibitor and/or angiotensin II receptor blocker); ACEI, angiotensin-converting enzyme inhibitor; ARB, angiotensin II receptor blocker.
